# AUCTSP: an improved biomarker gene pair class predictor

**DOI:** 10.1186/s12859-018-2231-1

**Published:** 2018-06-26

**Authors:** Dimitri Kagaris, Alireza Khamesipour, Constantin T. Yiannoutsos

**Affiliations:** 1 Department of Electrical and Computer Engineering, Southern Illinois University, 1230 Lincoln Drive, Carbondale, 62901 IL USA; 2 Department of Biostatistics, Indiana University School of Public Health, 410 West 10th Street, Suite 3000, Indianapolis, 46202 IN USA

**Keywords:** Microarray data analysis, Gene expression, Gene selection, Receiver operating characteristic (ROC) curve, AUC, Leukemia, Breast cancer, Ovarian cancer, Colon cancer, Prostate cancer, Diffuse large B-Cell lymphoma

## Abstract

**Background:**

The Top Scoring Pair (TSP) classifier, based on the concept of relative ranking reversals in the expressions of pairs of genes, has been proposed as a simple, accurate, and easily interpretable decision rule for classification and class prediction of gene expression profiles. The idea that differences in gene expression ranking are associated with presence or absence of disease is compelling and has strong biological plausibility. Nevertheless, the TSP formulation ignores significant available information which can improve classification accuracy and is vulnerable to selecting genes which do not have differential expression in the two conditions (“pivot" genes).

**Results:**

We introduce the AUCTSP classifier as an alternative rank-based estimator of the magnitude of the ranking reversals involved in the original TSP. The proposed estimator is based on the Area Under the Receiver Operating Characteristic (ROC) Curve (AUC) and as such, takes into account the separation of the entire distribution of gene expression levels in gene pairs under the conditions considered, as opposed to comparing gene rankings within individual subjects as in the original TSP formulation. Through extensive simulations and case studies involving classification in ovarian, leukemia, colon, breast and prostate cancers and diffuse large b-cell lymphoma, we show the superiority of the proposed approach in terms of improving classification accuracy, avoiding overfitting and being less prone to selecting non-informative (pivot) genes.

**Conclusions:**

The proposed AUCTSP is a simple yet reliable and robust rank-based classifier for gene expression classification. While the AUCTSP works by the same principle as TSP, its ability to determine the top scoring gene pair based on the relative rankings of two marker genes across *all* subjects as opposed to each individual subject results in significant performance gains in classification accuracy. In addition, the proposed method tends to avoid selection of non-informative (pivot) genes as members of the top-scoring pair.

**Electronic supplementary material:**

The online version of this article (10.1186/s12859-018-2231-1) contains supplementary material, which is available to authorized users.

## Background

Microarray data analysis is a high throughput method used to gain information about gene functions inside cells. This information is in turn used to detect the presence or absence of disease [1–3], and gain a better understanding of a disease mechanism [4].

A particularly useful application of microarray technology uses microarray data to detect the presence of disease by combining gene expression levels from a number of genes, to provide information on whether disease is present (classification) or the risk for the occurrence of disease in the future (prediction). While very complex classifiers can be constructed, a number of authors have expressed concern with the “black box” nature of these approaches [5] preferring simpler more interpretable classifiers in clinical applications [6, 7]. It is noted that the preference for the latter kind of classifiers should not be at the expense of their performance.

Classification involves, at its most fundamental level, a comparison between expression levels in one or more genes between two or more conditions (e.g., disease versus no disease). This comparison can be based on a fairly heuristic criterion (e.g., fold-change in gene expression [8]), or by using parametric or non-parametric statistical methods [9–12]. There are several advantages and disadvantages with each of these methods. For example, it is biologically plausible that genes with large differential expression levels should be part of a classification criterion. However, the fold-change criterion does not take gene expression variability into account and determining a cutoff is an arbitrary exercise [13]. On the other hand, parametric statistical methods, which are based on some variant of the t-test, provide some sense of one’s confidence on the gene expression difference, but frequently lose the intuitive appeal of heuristic methods like fold-change (e.g., when even small differences are statistically significant). In addition, parametric methods make strong and frequently untenable assumptions regarding the distribution of gene expression levels [13]. Non-parametric methods, which are based on ranking gene expression levels, are expected to lose some information because of the use of ranks instead of actual gene-expression data. However, such methods are robust to deviations from parametric assumptions [13], and are less vulnerable to biases arising from data normalization and other pre-processing steps [14], which are plausibly assumed to be rank-preserving [6, 7].

The fact that the TSP provides classifiers based on only two genes is also an attractive compromise in the so-called “bias-variance” tradeoff [15]. As a classifier’s performance is a combination of variance (random error) and bias (systematic error), in many cases, high-dimensional classifiers with low bias (due to good performance in the current sample) have large variances (i.e., poor precision) in new samples. By contrast, simpler (and thus more rigid) classifiers, while possibly having higher levels of bias, are less influenced by a specific sample and may have better overall performance (smaller variance) in multiple samples.

The simple TSP classifiers, it was hoped, would perform sufficiently well both in the current sample as well as in new samples. The TSP is a rank-based classifier in the sense that it uses the rankings of gene expression levels within a gene profile rather than the levels themselves, an approach with significant advantages due to the nonparametric nature of the classification technique. The central idea behind the TSP classifier is that it identifies two genes whose gene expression ranking changes between the two conditions under consideration. This change lends itself to a simple biological interpretation as an inversion of mRNA abundance of the two genes in the two conditions under consideration. The pair of genes selected by the TSP [6], referred to as the top scoring pair (TSP), is found by the following approach: Consider *G* genes which have been profiled by microarray analysis. Let *n*_1_ be the number of experiments from the first class with expression levels $Y_{i}=\{Y_{i,1}, Y_{i,2}, \cdots, Y_{i,n_{1}}\}\phantom {\dot {i}\!}$, and let *n*_2_ be the number of experiments from the second class with expression levels $\phantom {\dot {i}\!}Y_{i}=\{Y_{i,n_{1}+1}, Y_{i,n_{1}+2}, \cdots, Y_{i,n}\}$, where *n*=*n*_1_+*n*_2_. Given a pair of genes (*i*,*j*), 1≤*i*≠*j*≤*G*, the reversal score of the pair was defined in [6] as

1 $$ \Delta_{ij} = \left| P(Y_{i} > Y_{j}|C=1) - P(Y_{i} > Y_{j}|C=2)\right|  $$

where *P*(*Y*_*i*_>*Y*_*j*_|*C*=*m*) denotes the probability that the expression level of gene *i* is larger than the expression level of gene *j* in samples from class *C*, with *C* being equal to *m*=1,2. The score *Δ*_*ij*_ can be empirically approximated by the expression [6]

2 $$ D_{ij} =\left|\frac{\sum_{\ell=1}^{n_{1}}{{I}_{1}(Y_{i,\ell}>Y_{j,\ell})}}{n_{1}} - \frac{\sum_{\ell=n_{1}+1}^{n}{{I}_{2}(Y_{i,\ell}>Y_{j,\ell})}}{n_{2}}\right|  $$

where index *ℓ* indicates the *ℓ*th subject, 1≤*ℓ*≤*n* and *I*_*m*_(*Y*_*i*,*ℓ*_>*Y*_*j*,*ℓ*_)=1 if *Y*_*i*,*ℓ*_>*Y*_*j*,*ℓ*_ in class *m*=1,2, and 0 otherwise.

Obviously, the larger the *Δ*_*ij*_, the higher the probability that the expression levels of genes *i* and *j* have reverse relative rankings in the two groups, and it is exactly this property that is used for classification by the TSP. More specifically, let (*α*,*β*) be the pair of genes that yields the maximum score *Δ*_*αβ*_=max{*Δ*_*ij*_} (referred to as the Top Scoring Pair (TSP) [6]). Then the classification is performed as follows:

Assume that

3 $$P(Y_{\alpha} > Y_{\beta}|C=1) > P(Y_{\alpha} > Y_{\beta}|C=2)  $$

i.e.,

4 $$ \frac{\sum_{\ell=1}^{n_{1}}{{I}_{1}(Y_{\alpha,\ell}>Y_{\beta,\ell})}}{n_{1}} > \frac{\sum_{\ell=n_{1}+1}^{n}{{I}_{2}(Y_{\alpha,\ell}>Y_{\beta,\ell})}}{n_{2}}  $$

Then a new subject *s* whose measured expression levels for genes *a* and *b* are *Y*_*α*,*s*_ and *Y*_*β*,*s*_ respectively, will be classified as belonging to the first class if *Y*_*α*,*s*_>*Y*_*β*,*s*_, and to the second class otherwise.

The genes in the top scoring pair as selected by the TSP method may have a problem, as Lin et al. [5] also point out: the selected genes may not be a pair of genuinely up-regulated and down-regulated genes, but one of the selected genes in the pair happens to serve only as a reference or “pivot” gene that may lead to a high TSP score but a rather non-informative gene pair. Most researchers have used more complicated methods or selected more features in order to overcome the mentioned problems. In the proposed method we employ a simple statistic associated with the Receiver Operating Characteristic (ROC) curve that is commonly known as the Area Under the ROC curve (AUROC) or the Area Under the Curve (AUC), for short. The ROC curve and the AUC in particular have been widely used as a measure for microarray classification and other medical diagnostic tests (see, e.g., [16–23].

The proposed method, referred to as AUCTSP (AUC-based TSP), uses similar ideas as the TSP, thus benefiting from the simplicity of the TSP approach, but enhances TSP by making the resulting classifier less prone to overfitting, achieving higher classification accuracy and avoiding the selection of pivot genes as members of the top scoring pair of genes.

## Methods

In this manuscript we propose the AUCTSP, a classifier that works according to the same principle as TSP but differs from the latter in that the probabilities that determine the top scoring pair are computed based on the relative rankings of the two marker genes across all subjects instead of within each individual subject. Although the classification is still done on an individual-subject basis, consideration of all subject data in the estimation of the ranking reversals results in a classifier with higher accuracy. This performance superiority of AUCTSP over TSP is demonstrated through simulations and case studies (see “Results” section) involving classification in ovarian, leukemia, colon, prostate and breast cancers and diffuse large b-cell lymphoma.

### The proposed AUCTSP classifier

The score that TSP computes is based on the probability *P*(*Y*_*i*_>*Y*_*j*_|*C*=*m*) that the expression level of gene *i* is larger than the expression level of gene *j* in samples from the *m*-th class, *m*=1,2. This probability was approximated in [6] by the proportion of individuals of class *m* with higher expression level in gene *i* than in gene *j* out of all individuals in class *m*, i.e., by the probability

5 $$ P_{\text{TSP}}(Y_{i} > Y_{j}|C=m)=\frac{\sum_{\ell=1}^{n_{m}}{{I}_{m}(Y_{i,\ell}>Y_{j,\ell})}}{n_{m}}   $$

We propose to approximate the original probability *P*(*Y*_*i*_>*Y*_*j*_|*C*=*m*) by the probability that a randomly chosen individual from class *m* has an expression level for gene *i* that is larger than that of a randomly chosen individual from class *m* (*m*=1,2) for gene *j*.

The estimate of the original probability *P*(*Y*_*i*_>*Y*_*j*_|*C*=*m*) in the proposed AUCTSP method is given by

$$P_{\text{AUCTSP}}(Y_{i}>Y_{j}|C=m)=$$

6 $$ \frac{\sum_{k=1}^{n_{m}}\sum_{\ell=1}^{n_{m}}{{I}(Y_{i,k}>Y_{j,\ell})}}{n_{m}^{2}}   $$

The numerator in Eq. 6 denotes the sum over all samples *k*, 1≤*k*≤*n*_*m*_, of the number of times that the expression level of gene *i* in sample *k* is larger than the expression level of gene *j* in some other sample *ℓ*≠*k*,1≤*ℓ*≤*n*_*m*_, from the same class *m* (*m*=1 or 2).

The probability *P*_AUCTSP_ can be calculated by the Area Under the ROC Curve (AUC) [23]. The AUC statistic has been used extensively in diagnostic test validation [18–20, 22, 23] and gene feature selection [21] in two-group settings. In our case here, group 1 is taken to be the set of expression levels of gene *i* in class *m*, and group 2 is taken to be the set of expression levels of gene *j* in *the same* class *m*. It is well established that, for independent samples, the AUC statistic is the minimum-variance unbiased estimate of *P*(*X*>*Y*) [24]. In correlated samples (as we have here, since the gene expression levels are measured on the same individual *i*=1,2,⋯,*n*_*m*_ for *m*=1,2), it is expected that *P*_AUCTSP_ is still an unbiased estimate of *P*(*X*>*Y*) and should generate more precise estimates of the probability *P*(*Y*_*i*_>*Y*_*j*_|*C*=*m*) compared to *P*_TSP_, unless the correlation of gene expression levels between genes *i* and *j* in the same individual is too high (thus leading to an inflated variance of the AUC-based estimator). In addition, the AUCTSP classifier, which is based on a summary measure derived from *all* subjects (compared to the single-subject approach in the TSP), has the potential to yield a top scoring pair that is less susceptible to the specific training data, thus further avoiding overfitting compared to the TSP. The better performance of AUCTSP is corroborated by our experimental results.

We highlight the following two points about our use of the AUC statistic in the proposed method: (i) the AUC statistic is traditionally applied on two groups one of which is the “healthy” and the other one the “diseased,” whereas in our method we apply it on gene expression profiles from the same (“healthy” or “diseased”) group; (ii) although the *P*_AUCTSP_ is obtained from *all* subjects, the classification rule that we obtain in the AUCTSP classifier is still applied on the expression levels of the marker genes from the *same* single subject, exactly as in the TSP classifier.

To elucidate the intuition behind the AUCTSP classifier, consider the following example. Assume that the expression levels of a gene *A* for 5 healthy subjects are as given in Table 1. The probability that the expression level of *A* is less than the level of *B* in the healthy subjects is 5/5 = 1 while the probability that the level of *A* is less than the level of *B* in the diseased subjects is 0, yielding an overall TSP score $D^{\tiny \mathrm {TSP}}_{AB}= 1$. Contrast the above with the situation involving two other genes, *C* and *D* (Table 1). The probability that the expression level of *C* is less than the level of *D* in the healthy subjects is 4/5 = 0.8, while the probability that the expression level of *C* is less than the level of *D* in the diseased subjects is 1/5 = 0.2. This yields an overall TSP score $D^{\tiny \text {TSP}}_{CD}=0.6$, which is less than the score of pair *A* and *B*, and consequently the pair *C* and *D* would be discarded by the TSP. However, the *distributions* of the expression levels of *C* and *D* in the healthy (and the diseased) subjects exhibit greater separation than those for *A* and *B* and thus, using genes *C* and *D* for classification is arguably preferable.

**Table 1:** Gene expression levels in two genes

Healthy		Diseased		Healthy		Diseased	
Gene *A*	Gene *B*	Gene *A*	Gene *B*	Gene *C*	Gene *D*	Gene *C*	Gene *D*
11	12	32	31	10	20	42	31
21	22	34	33	12	23	43	33
23	24	36	35	15	25	45	35
25	26	38	37	17	27	47	37
27	28	40	39	19	18	39	41

The above intuitive preference for pair (*C*,*D*) is supported by the score derived for these two genes according to the proposed AUCTSP approach. The non-parametric estimate of the AUC for pair (*C*, *D*) on the healthy subjects is 24/25 = 0.96, and on the diseased subjects it is 1/25 = 0.04. This yields an overall AUCTSP score of $D^{\tiny \text {AUCTSP}}_{CD}=0.92$, while the corresponding AUCTSP score for the (*A*,*B*) gene pair is $D^{\tiny \text {AUCTSP}}_{AB}=15/25 - 10/25 = 0.2$ and, therefore, the (*C*,*D*) pair is preferred over(*A*,*B*) by the proposed approach. We note here that the claim about the greater separation of the gene expression distributions is not based in any way on the actual values of the data, only on their ranking. This in turn means that the proposed method will be robust in selecting the top scoring pair and will not be affected by outliers in the gene expression data and will also be invariable to any rank-preserving normalization technique.

## Results

The AUCTSP classifier was implemented in the C programming language. The evaluation of the methodology was based on (i) simulations and (ii) case studies.

### Simulations

We compared the estimations given by TSP (Eq. 5) and AUCTSP (Eq. 6) for the probability *P*(*X*>*Y*) involved in the computation of the TSP and AUCTSP scores. We generated random expression levels for “genes” *X* and *Y* from normal distributions with different combinations of mean *μ* and deviation *σ* for different sample sizes, where *μ*_*X*_ is greater than or equal to *μ*_*Y*_ in all of the simulated cases. In this case, the probability *P*(*X*>*Y*) is given by the detectability index *A*_*z*_ defined by Metz et al. [22] as:

7 $$ A_{z} = P(X > Y) = \Phi\left (\frac{\frac{|\mu_{X} - \mu_{Y}|}{\sigma_{X}}}{\sqrt{1 +\left (\frac{\sigma_{Y}}{\sigma_{X}}\right)^{2}}}\right)  $$

where *Φ*() denotes the cumulative distribution function (CDF) of the standard normal distribution and *μ*_*X*_, *σ*_*X*_, and *μ*_*Y*_, *σ*_*Y*_ denote the mean and standard deviation of the assumed normal distributions for *X* and *Y*, respectively.

The cases chosen for comparison are two normal distributions with:

(i) small means (*μ*_*X*_=1, *μ*_*Y*_=0) with small variances (*σ*_*X*_=1, *σ*_*Y*_=1);
(ii) small means (*μ*_*X*_=1, *μ*_*Y*_=0) with large variances (*σ*_*X*_=3, *σ*_*Y*_=3);
(iii) large means (*μ*_*X*_=5, *μ*_*Y*_=0) with small variances (*σ*_*X*_=1, *σ*_*Y*_=1);
(iv) large means (*μ*_*X*_=5, *μ*_*Y*_=0) with large variances (*σ*_*X*_=3, *σ*_*Y*_=3);
(v) equal small means (*μ*_*X*_=1, *μ*_*Y*_=1) with a small variance for one distribution (*σ*_*X*_=1) and a large variance for the other distribution (*σ*_*Y*_=3);
(vi) equal large means (*μ*_*X*_=5, *μ*_*Y*_=5) with a small variance for one distribution (*σ*_*X*_=1) and a large variance for the other distribution (*σ*_*Y*_=3).

The results for different sample sizes *N*=10,20,30,40 are shown in Table 2. Columns 4 and 5 show the estimates of probability *P*(*X*>*Y*) obtained by TSP and AUCTSP over 1000 random trials. The theoretical probability *A*_*z*_=*P*(*X*>*Y*) (see Eq. 7) is shown in the last column. With bold, we show the value that is closer to the theoretical value *A*_*z*_. As can be seen, for the cases where both simulated gene expression distributions have equal variances (cases i-iv), the AUCTSP and TSP estimates are virtually identical and are very close to the theoretical probability even for small sample sizes. In the two cases where the variance in one of the genes is greater (cases v-vi), both estimators do poorly for small sample size *N* and improve with increasing *N*, but the AUCTSP is always closer to the target quantity *A*_*z*_.

**Table 2:** Simulation results on estimation of *P*(*X*>*Y*) by TSP and AUCTSP

Gene X	Gene Y	*N*	TSP	AUCTSP	*A*_*z*_
N(1,1)	N(0,1)	10	0.763	**0.762**	0.760
		20	0.762	**0.761**	0.760
		30	0.759	**0.760**	0.760
		40	0.759	**0.760**	0.760
N(1,3)	N(0,3)	10	0.595	**0.594**	0.592
		20	0.594	**0.593**	0.592
		30	0.594	**0.593**	0.592
		40	0.593	**0.592**	0.592
N(5,1)	N(0,1)	10	**0.998**	**0.998**	0.999
		20	**0.998**	**0.998**	0.999
		30	**0.998**	**0.998**	0.999
		40	**0.998**	**0.998**	0.999
N(5,3)	N(0,3)	10	0.883	**0.882**	0.878
		20	0.881	**0.880**	0.878
		30	0.880	**0.879**	0.878
		40	0.880	**0.879**	0.878
N(1,1)	N(1,3)	10	0.619	**0.610**	0.500
		20	0.587	**0.581**	0.500
		30	0.572	**0.564**	0.500
		40	0.563	**0.557**	0.500
N(5,1)	N(5,3)	10	0.616	**0.610**	0.500
		20	0.585	**0.575**	0.500
		30	0.570	**0.563**	0.500
		40	0.559	**0.554**	0.500

The estimates of *P*(*X*>*Y*) closer to *A*_*z*_ are marked in bold

Next, we compared the capability of TSP and AUCTSP to identify the single informative pair of genes in the midst of other non-informative genes. For this purpose, we generated random normal expression levels for *N* “genes” from *n*_1_ “healthy” individuals and *n*_2_ “diseased” individuals, for all combinations of *N*=100, 200 and *n*_1_=*n*_2_=20, 40. In all these simulations the genes numbered 1 and 2 carry the differentiating information between the healthy and diseased groups, represented by normal distributions (NH() for the “healthy” and ND() for the “diseased”) that are different from N(0,1), as shown in Table 3. All remaining genes other than 1 and 2 have expression levels obtained from the same “non-informative” distribution N(0,1). The efficacy of each classifier is measured by how many times it is able to identify the pair of genes (1,2) as the top scoring pair. The results (as averages over 1000 simulations) are shown in Table 3. The rows correspond to cases exploring the effect of increasing variance and increasing differences in the means of the expression level distributions. As can be observed, the AUCTSP consistently outperforms the TSP, in some cases dramatically, even for small sample sizes.

**Table 3:** Simulation results for the ability of AUCTSP and TSP to identify the most informative gene pair

Gene 1	Gene 2	N=100 *n*_1_=*n*_2_=20		N=100 *n*_1_=*n*_2_=40		N=200 *n*_1_=*n*_2_=20		N=200 *n*_1_=*n*_2_=40	
		TSP	AUCTSP	TSP	AUCTSP	TSP	AUCTSP	TSP	AUCTSP
NH(0,1) ND(1,1)	NH(1,1) ND(0,1)	23.4	51.2	58.8	93.2	15.4	39.8	45.4	89.7
NH(-1,1) ND(1,1)	NH(1,1) ND(-1,1)	69.1	98.9	97.7	99.9	57.8	97.2	94.0	99.9
NH(-2,1) ND(2,1)	NH(2,1) ND(-2,1)	91.6	99.9	97.6	99.9	92.7	99.8	95.7	99.9
NH(-2,2) ND(2,2)	NH(2,2) ND(-2,2)	48.2	93.2	80.2	99.9	38.3	91.4	71.4	99.9

### Case studies

We evaluated the performance of the AUCTSP classifier over the TSP classifier in 8 publicly available datasets:

(i) Ovarian Cancer (Pepe et al., 2003 [17]) dataset which consists of 1536 genes with expression levels from 23 healthy and 30 diseased subjects;
(ii) Acute Leukemia (Golub et al., 1999 [25]) dataset which consists of 3571 human genes with expression levels from 25 cases of acute myeloid (aka myelogenous) leukemia (AML) and 47 cases from acute lymphoblastic (aka lymphocytic) leukemia;
(iii) Breast Cancer - Estrogen Receptor (ER) status (West et al., 2001 [26]) dataset which consists of the expression levels of 7129 genes in 49 tissues separated into two groups of 25 positive and 24 negative tissues based on the estrogen receptor (ER) status;
(iv) Breast Cancer - Lymph Node (LN) status (West et al., 2001 [26]) dataset which consists of the expression levels of 7129 genes in 49 tissues separated into two groups of 24 positive and 25 negative tissues based on the lymph node (LN) status;
(v) Diffuse Large B-Cell Lymphoma (DLBCL) to predict patient outcome (Alizadeh et al., 2000 [27]) dataset which consists of the expression levels of 7129 genes in 32 cured samples and 26 fatal or refractory disease samples.
(vi) DLBCL versus Follicular Lymphoma (FL) (Alizadeh et al., 2000 [27]) dataset which consists of the expression levels of 7129 genes in 58 DLBCL samples and 19 FL samples;
(vii) Colon Cancer (Alon et al., 1999 [28]) dataset which consists of the expression levels of 2000 genes from 40 subjects diagnosed with colon cancer and 22 healthy subjects;
(viii) Prostate cancer (Singh et al., 2002 [29]) dataset which consists of the expression levels of 12533 genes from 52 subjects diagnosed with prostate cancer and 50 healthy subjects.

### Top scoring pairs selected by TSP and AUCTSP

For each of these datasets, we applied AUCTSP and TSP and identified the top-scoring pairs obtained by AUCTSP and TSP. The selected pairs of genes are shown in Table 4 and the gene legend is shown in Table 5.

**Table 4:** Top scoring pairs of genes under TSP and AUCTSP

			Score	
Dataset	Method	Gene pair	TSP	AUCTSP
OVARIAN	TSP	[PKM2, OVGP1]	0.900	0.675
	AUCTSP	[IRS1, OVGP1]	0.833	0.826
LEUKEMIA	TSP	[SPTAN1, CD33]	0.979	0.938
	TSP	[ARHGAP45, ZYX]	0.979	0.770
	TSP	[PCDHGC3, ZYX]	0.979	0.855
	AUCTSP	[SPTAN1, CD33]	0.979	0.938
BREAST-ER	TSP	[MUC2, ESR1]	0.918	0.812
	TSP	[JAK3, ESR1]	0.918	0.791
	TSP	[GNB3, ESR1]	0.918	0.804
	TSP	[HARS2, ESR1]	0.918	0.834
	TSP	[ERF, ESR1]	0.918	0.822
	AUCTSP	[CTSC, ESR1]	0.878	0.891
BREAST-LN	TSP	[BP1CR, GYPB]	0.838	0.675
	AUCTSP	[BP1CR, KRT31]	0.717	0.765
	TSP	[FABP3, ACVR1B]^b^	0.716	0.531
	AUCTSP	[GYPB, ACVR1B]^b^	0.633	0.615
DLBCL	TSP	[PDE4B, GPR12]	0.596	0.414
	AUCTSP	[POLR2J, PTGER4]	0.341	0.46
DLBCL-FL	TSP	[YWHAZ, SNRPB]	0.983	0.727
	AUCTSP	[FCGR1A, NEO1]	0.759	0.83
COLON	TSP	[VIP, DARS]	0.879	0.637
	AUCTSP	[MYH9, HNRNPA1]	0.759	0.724
PROSTATE	TSP	[CFD, ENO1]	0.901	0.693
	AUCTSP	[CFD, NUMB]	0.882	0.883

^a^indicates the selected TSP gene pair by [7] to break the tie for pairs with equal TSP scores

^b^indicates the selected pair of genes by TSP and AUCTSP after removing the genetically modified gene BP1CR (see [32, 33]) from the dataset

**Table 5:** Gene legend

Data set	Gene ID	Gene acronym	Gene description
OVARIAN	g47	IRS1	Insulin Receptor Substrate 1
	g93	OVGP1	Oviductal Glycoprotein 1
	g1202	PKM2	Pyruvate Kinase, Muscle
LEUKEMIA	D86976	ARHGAP45	Rho GTPase Activating Protein 45
	J05243	SPTAN1	Spectrin Alpha, Non-Erythrocytic 1
	L11373	PCDHGC3	Protocadherin Gamma Subfamily C, 3
	M23197	CD33	CD33 Molecule
	X95735	ZYX	Zyxin
BREAST-ER	L21998	MUC2	Mucin 2
	U09607	JAK3	Janus Kinase 3
	U15655	ERF	ETS2 Repressor Factor
	U18937	HARS2	Histidyl-TRNA Synthetase 2, Mitochondrial
	U47931	GNB3	G Protein Subunit Beta 3
	X03635	ESR1	Estrogen Receptor 1
	X87212	CTSC	Cathepsin C
BREAST-LN	AFFX-CreX-3	BP1CR	Bacteriophage P1 Cre Recombinase
	X82634	KRT31	Keratine 31
	J02982	GYPB	Glycophorin B
	M18079	FABP3	Fatty Acid Binding Protein 3
	X15357	ACVR1B	Activin A Receptor Type 1B
DLBCL	K03008	POLR2J	RNA Polymerase II Subunit J
	L20971	PDE4B	Phosphodiesterase 4B
	L28175	PTGER4	Prostaglandin E Receptor 4
	U18548	GPR12	G Protein-Coupled Receptor 12
DLBCL-FL	D78134	YWHAZ	Tyrosine 3-Monooxygenase/ Tryptophan 5-Monooxygenase Activation Protein Zeta
	M63835	FCGR1A	Fc Fragment Of IgG Receptor Ia
	U61262	NEO1	Neogenin 1
	X17567	SNRPB	Small Nuclear Ribonucleoprotein Polypeptides B and B1
COLON	Hsa.37937	MYH9	Myosin Heavy Chain 9
	Hsa.8010	HNRNPA1	Heterogeneous Nuclear Ribonucleoprotein A1
	Hsa.2097	VIP	Vasoactive Intestinal Peptide
	Hsa.601	DARS	Aspartyl-TRNA Synthetase
PROSTATE	40282_s_at	CFD	Complement Factor D
	2035_s_at	ENO1	Enolase 1
	37693_at	NUMB	NUMB, Endocytic Adaptor Protein

Table 4 reports also (for informational purposes) the score that the selected pair by TSP and AUCTSP receives under the opposite classifier (AUCTSP and TSP, respectively). For example, the pair selected by TSP for the ovarian cancer dataset has a TSP score of 0.9 but it receives a score of 0.675 under AUCTSP, whereas the AUCTSP score of the pair selected by AUCTSP is 0.826, while the score given to it by TSP is 0.833. This shows that pairs selected by TSP may have significantly lower scores under AUCTSP.

The biological relevance of the selected genes was found by consulting the GENECARDS database [30] and the VarElect NGS Phenotyper [31]. All of the genes identified by AUCTSP have been reported in the existing literature to be indeed related to the corresponding disease, whereas some of the genes identified by TSP such as DARS for colon cancer have not been reported to be related. A full description of the biological findings on the genes selected by AUCTSP and TSP is given in the Additional file 1. The histograms of the selected genes are also given in the Additional file 2.

We also note that for the datasets examined, AUCTSP resulted in no ties, whereas TSP frequently selected multiple pairs of genes having the same highest TSP score (3 such pairs in the Leukemia dataset and 5 pairs in the Breast-ER dataset). We have identified the gene pair ultimately chosen by the TSP after applying the tie-breaking rule proposed by Geman et al. [6] with an asterisk (“*”) in Table 4. (For the case of the Breast-LN dataset, both the AUCTSP and TSP resulted in selecting a genetically modified gene (“Bacteriophage P1 Cre recombinase”) [32, 33] as member the top-scoring pair. The pair of genes selected by the AUCTSP and TSP after eliminating this gene from the dataset are marked with (“**”) in Table 4).

Furthermore, in order to check how far the selected genes (by either method) are from being non-informative “pivot” genes, we computed for each gene *g* the probability *P*_*g*_=*P*(*g*∈*C*_1_>*g*∈ *C*_2_) that the expression levels of *g* in class *C*_1_ are greater than the expression levels of *g* in class *C*_2_, where *C*_1_,*C*_2_ are the two classes in the corresponding dataset. A value of *P*_*g*_ close to 0.5 means that the gene is strongly non-informative. A value of *P*_*g*_ close to 1 or close to 0 means that the gene is strongly informative. For the case where the value of *P*_*g*_ is close to 0, we can simply inverse the ROC curve to compute the probability *P*(*g*∈*C*_1_<*g*∈ *C*_2_), so that all informative genes are indicated by values of *P*_*g*_ close to 1. The computation of *P*_*g*_ was done by computing the AUC of the ROC curve corresponding to the expression values of gene *g* in classes *C*_1_ and *C*_2_. The results are shown in Table 6. The *P*_*g*_ values for each member of a selected pair are shown in column 4, whereas column 5 shows the corresponding values $\hat {P}_{g}$ if the ROC curve has to be inverted so that values closer to 1 indicate more informative genes. As can be seen, the genes selected by AUCTSP have better deviation from the 0.5 value of a non-informative gene in almost every case.

**Table 6:** Deviation of the genes selected by TSP and AUCTSP from the non-informative “pivot” gene

Dataset	Method	Gene Pair	($P_{g_{1}},P_{g_{2}}$)	($\hat {P}_{g_{1}},\hat {P}_{g_{2}}$)
OVARIAN	TSP	(PKM2, OVGP1)	(0.16, 0.03)	(0.84, 0.97)
	AUCTSP	(IRS1, OVGP1)	(0.84, 0.03)	(0.84, 0.97)
LEUKEMIA	TSP	(SPTAN1, CD33)^a^	(0.05, 0.99)	(0.95, 0.99)
	TSP	(ARHGAP45, ZYX)	(0.61, 0.02)	(0.61, 0.98)
	TSP	(PCDHGC3, ZYX)	(0.63, 0.02)	(0.63, 0.98)
	AUCTSP	(SPTAN1, CD33)	(0.95, 0.01)	(0.95, 0.99)
BREAST-ER	TSP	(MUC2, ESR1)^a^	(0.72, 0.04)	(0.72, 0.96)
	TSP	(JAK3, ESR1)	(0.66, 0.04)	(0.66, 0.96)
	TSP	(GNB3, ESR1)	(0.56, 0.04)	(0.56, 0.96)
	TSP	(HARS2, ESR1)	(0.57, 0.04)	(0.57, 0.96)
	TSP	(ERF, ESR1)	(0.58, 0.04)	(0.58, 0.96)
	AUCTSP	(CTSC, ESR1)	(0.91, 0.04)	(0.91, 0.96)
BREAST-LN	TSP	(FABP3, ACVR1B)	(0.60, 0.69)	(0.60, 0.69)
	AUCTSP	(GYPB, ACVR1B)	(0.14, 0.69)	(0.86, 0.69)
DLBCL	TSP	(PDE4B, GPR12)	(0.73, 0.32)	(0.73, 0.68)
	AUCTSP	(POLR2J, PTGER4)	(0.30, 0.72)	(0.70, 0.72)
DLBCL-FL	TSP	(YWHAZ, SNRPB)	(0.80, 0.10)	(0.80, 0.90)
	AUCTSP	(FCGR1A, NEO1)	(0.06, 0.84)	(0.94, 0.84)
COLON	TSP	(VIP, DARS)	(0.82, 0.16)	(0.82, 0.84)
	AUCTSP	(MYH9, HNRNPA1)	(0.89, 0.24)	(0.89, 0.76)
PROSTATE	TSP	(CFD, ENO1)	(0.91, 0.27)	(0.91, 0.73)
	AUCTSP	(CFD, NUMB)	(0.91, 0.04)	(0.91, 0.96)

^a^indicates the selected TSP gene pair by [7] to break the tie for pairs with equal TSP scores

### Classifier performance of AUCTSP vs. TSP

We also compared the performance of the proposed AUCTSP classifier vs. the TSP classifier in terms of accuracy for predicting the correct status of subjects in a “testing” set after the classification rule (i.e., the top-scoring pair and its associated probabilities under AUCTSP and TSP, respectively) is obtained from a “training” set.

For each of the eight datasets in our case study, we generated several training sets and testing sets, by randomly picking a percentage *p* of subjects to form the training set and using the remaining *q*=1−*p* percentage of subjects as the testing set, for different values of *q*=1*%*,5*%*,10*%*,15*%*,20*%*,30*%*. The actual size of the testing set was set to ⌈*N*·*q*⌉, where *N* is the size of the dataset, and the set of the training set was set to *N*−⌈*N*·*q*⌉. Our intention was to see how AUCTSP and TSP behave as the training set decreases, i.e., how well AUCTSP and TSP can “generalize” their classification rule. Each test was repeated for 1000 trials and the average of the classifier accuracy (i.e., the ratio of the sum of the true positive and true negative test cases identified by the classification rule obtained from the training set over the total number of test cases) was calculated over these trials for each training set.

**Fig. 1:**
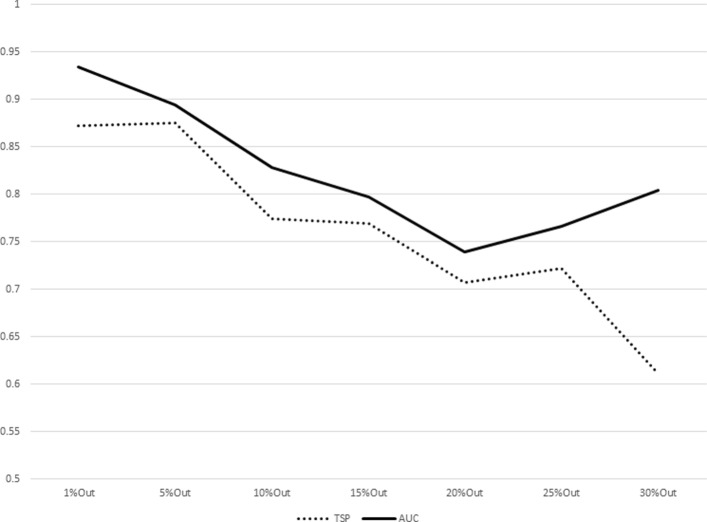
Comparison of TSP vs. AUCTSP classification accuracy for different sizes of training sets: OVARIAN dataset

**Fig. 2:**
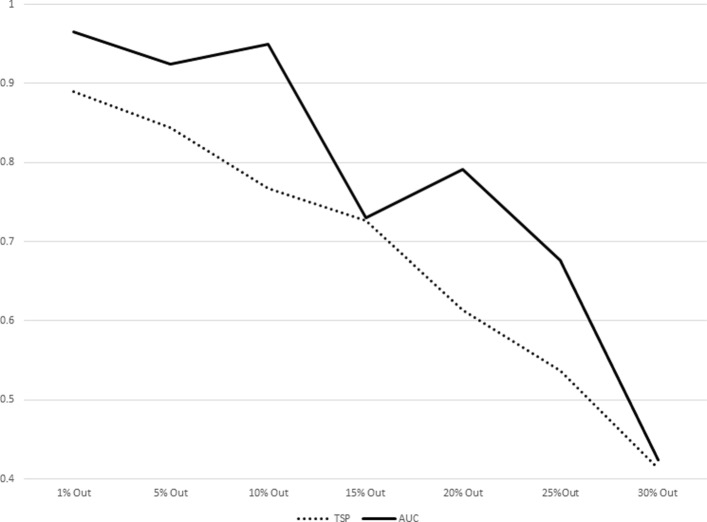
Comparison of TSP vs. AUCTSP classification accuracy for different sizes of training sets: COLON dataset

**Fig. 3:**
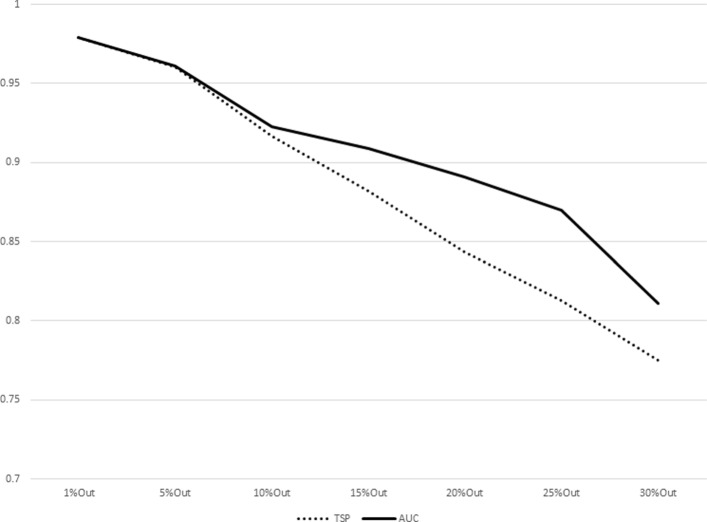
Comparison of TSP vs. AUCTSP classification accuracy for different sizes of training sets: LEUKEMIA dataset

**Fig. 4:**
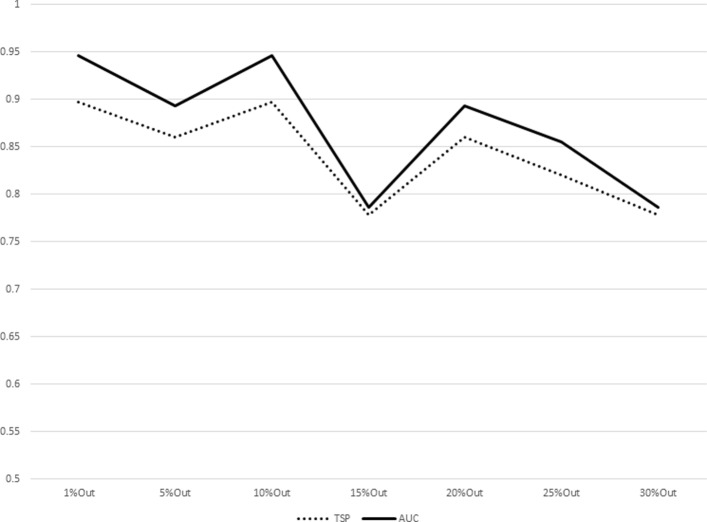
Comparison of TSP vs. AUCTSP classification accuracy for different sizes of training sets: BREAST-LN dataset

**Fig. 5:**
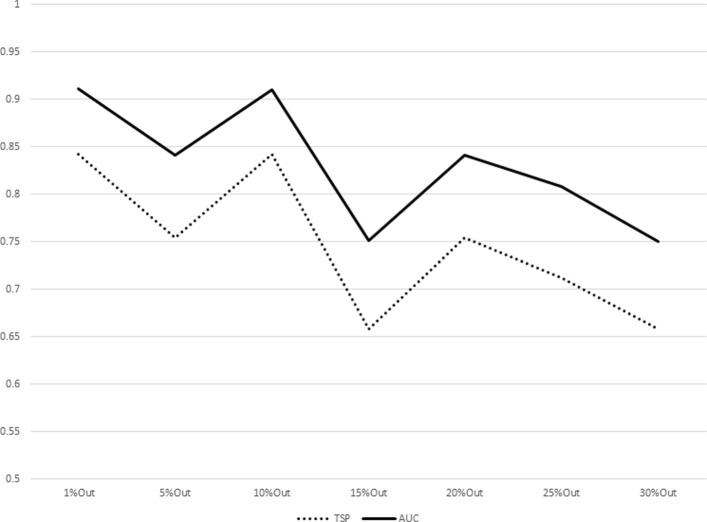
Comparison of TSP vs. AUCTSP classification accuracy for different sizes of training sets: BREAST-ER dataset

**Fig. 6:**
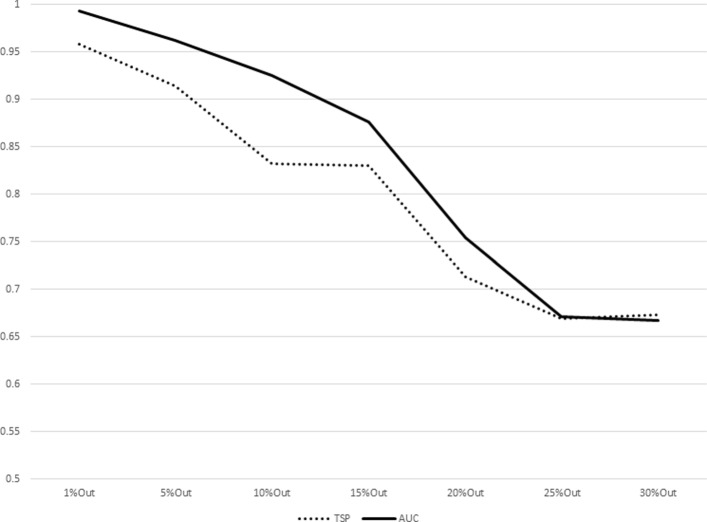
Comparison of TSP vs. AUCTSP classification accuracy for different sizes of training sets: DLBCL-FL dataset

**Fig. 7:**
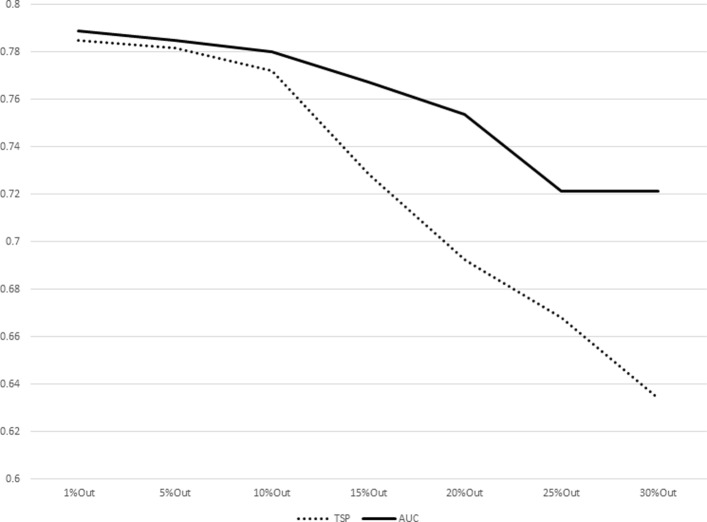
Comparison of TSP vs. AUCTSP classification accuracy for different sizes of training sets: DLBCL dataset

**Fig. 8:**
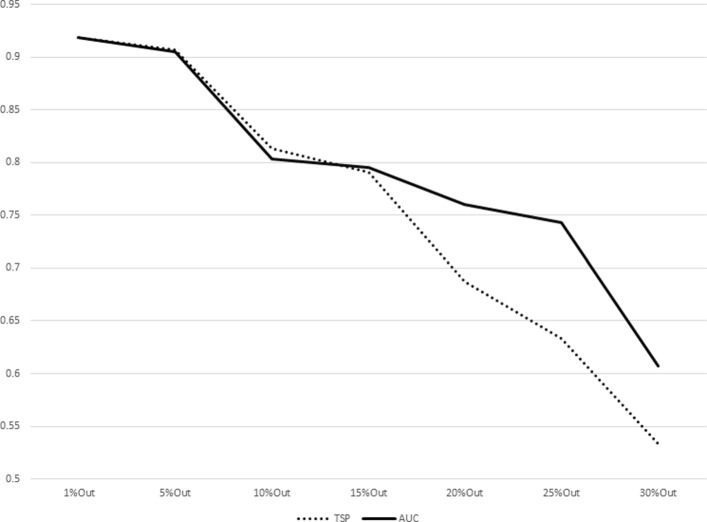
Comparison of TSP vs. AUCTSP classification accuracy for different sizes of training sets: PROSTATE dataset

The results for increasing sizes of test sets (equivalently, decreasing sizes of training sets) as percentages of subjects left out from the original dataset are shown in Table 7. The plot representations of the results listed in Table 7 are given in Figs. 1, 2, 3, 4, 5, 6, 7 and 8. These results show that the AUCTSP method performs better in terms of classification accuracy than the TSP method. The results indicate that the AUCTSP classifier is able indeed to identify useful marker genes from small training sets, in accordance with the “generalization” capability of the AUC statistic.

**Table 7:** Comparison of classifier accuracy by TSP and AUCTSP for decreasing size of training set

Test set fraction	OVARIAN		LEUKEMIA		COLON		BREAST-LN		BREAST-ER		DLBCL		DLBCL-FL		PROSTATE	
	TSP	AUCTSP	TSP	AUCTSP	TSP	AUCTSP	TSP	AUCTSP	TSP	AUCTSP	TSP	AUCTSP	TSP	AUCTSP	TSP	AUCTSP
1%	87.18	93.39	97.89	97.89	88.98	96.59	89.76	94.66	84.26	91.07	78.50	78.88	95.80	99.30	91.90	91.90
5%	87.48	89.43	96.02	96.12	84.45	92.45	86.03	89.35	75.40	84.11	78.20	78.50	91.46	96.23	90.70	90.50
10%	77.43	82.78	91.64	92.27	76.76	95.01	89.76	94.66	84.26	91.06	77.20	78.02	83.18	92.49	81.34	80.37
15%	76.96	79.7	88.2	90.9	72.71	73.02	77.85	78.6	65.84	75.07	72.84	76.73	83.02	87.57	79.10	79.50
20%	70.71	73.95	84.32	89.1	61.39	79.15	86.03	89.35	75.39	84.10	69.23	75.35	71.30	75.45	68.70	76.06
25%	72.2	76.6	81.27	87	53.75	67.65	82.05	85.48	71.20	80.80	66.79	72.11	66.87	67.14	63.30	74.35
30%	61.15	80.38	77.53	81.1	41.38	42.39	77.85	78.6	65.84	75.06	63.41	72.13	67.35	66.74	53.30	60.7

## Discussion

AUCTSP maintains the basic advantages of TSP namely the data-driven and parameter-free machine learning features that resolve the parameter tuning issue without making any assumptions about the data used, as well as the production of easily interpretable classification rules. AUCTSP, however, improves TSP by avoiding overfitting and suffering less from small sample sizes, due to the fact that every sample is compared to all other samples in the same class rather than on only a single sample by sample comparison as in TSP. In addition, AUCTSP tends to avoid selection of non-informative pivot genes, which are a known problem of TSP. Concerning selection of genes whose over-expression or under-expression is due to reasons unrelated to the disease in question, we note that this is less likely to create a problem since pairs of genes rather than single genes have to be affected in that way.

Finally, we note that AUCTSP can be extended to select a number of *k*>1 pairs of genes, with the classification being made according to a majority voting rule among those *k* pairs of genes, as was done in [7], or to find triplets instead of pairs of genes as was done in [5]. As a non-parametric based technique, AUCTSP can also have potential benefits in areas such as RNA sequence analysis (see, e.g. [34]), but this extension is left for future work.

## Conclusion

In this paper, we have proposed the AUCTSP, a simple yet reliable and robust rank-based classifier for gene expression classification. AUCTSP works according to the same principle as TSP but differs from the latter in that the probabilities that determine the top scoring pair are computed based on the relative rankings of the two marker genes across *all* subjects as opposed to for *each* individual subject. Results of calculating and comparing the AUCTSP and TSP probabilities for synthetic data as well as 8 publicly available datasets demonstrate the better performance of AUCTSP over TSP.

## Additional files

Additional file 1 Biological relevance of the selected gene pairs. A full description of the biological findings on the genes selected by AUCTSP and TSP is given. (PDF 112 kb)

Additional file 2 Histograms of the selected genes. The histograms of all the genes selected by AUCTSP and TSP are given. (PDF 294 kb)

## Abbreviations

AUC: Area under the (ROC) curve
AUCTSP: AUC-based TSP
AUROC: Area under the ROC (curve)
ROC: Receiver operating characteristic (curve)
TSP: Top scoring pair
