# Supplementary material for: AUCTSP: an improved biomarker gene pair class predictor
Source: BMC Bioinformatics. 2018 Jun 26;19:244. doi: 10.1186/s12859-018-2231-1 (PMC6020231; doi:10.1186/s12859-018-2231-1)
Supplement: Supplementary file 1 — Biological relevance of the selected gene pairs. A full description of the biological findings on the genes selected by AUCTSP and TSP is given. (PDF 112 kb) [file 12859_2018_2231_MOESM1_ESM.pdf]

Supplemental Material:

Biological relevance of the selected gene pairs

In the following, we summarize the evidence we have found on the biological relevance of the top-scoring gene pairs selected by AUCTSP and TSP, as potential composite biomarkers of various types of cancer in the datasets we used as case studies in the main paper.

Ovarian Cancer dataset: The AUCTSP method selected "Oviductal Glycoprotein 1" (OVGP1) and "Insulin Receptor Substrate 1" (IRS1) as the most informative pair of genes for diagnosing ovarian cancer. The expression level of OVGP1 has been shown to be higher in ovarian cancer patients, depending on both the ovarian cancer tumor histotypes and the stage of the tumor [1]. The expression level of IRS1 has been shown to be significantly lower in ovarian cancer patients [2]. The TSP method selected OVGP1 and "Pyruvate Kinase Muscle" (PKM) from the same dataset as a potential pair of biomarkers for ovarian cancer. PKM has been shown to have significant contribution to the treatment resistance in ovarian cancer cells [3, 4] and the development of ovarian cancer [5, 6].

Acute Leukemia dataset: The AUCTSP method selected "CD33 Molecule" (CD33) and "Spectrin Alpha, Non-Erythrocytic 1" (SPTAN1) as the most informative pair of genes for distinguishing Acute Myeloid Leukemia from Acute Lymphoid Leukemia. Although the way CD33 expression is regulated still remains unclear, CD33 is known to be informative for separating lymphoid from myeloid lineage cells [7]. On the other hand, loss of SPTAN1 has been shown to be associated with the development of both myeloid [8] as well as lymphoid leukemia [9]. The TSP method selected three pairs of genes with the same highest TSP score: CD33 and SPTAN1, "Rho GTPase Activating Protein 45" (ARHGAP45) and "Zyxin" (ZYX), and "Protocadherin 43" (PCDHGC3) and ZYX. ARHGAP45 is related to acute leukemia indirectly by being encoded through the "Protein Tyrosine Phosphatase, Non-Receptor Type 11" (SHP2) under certain circumstances [10]. SHP2 has been reported to relate to human solid tumors and adult acute myelogenous leukemia [11]. On the other hand, ZYX has been reported to be strongly associated with "Vasodilator-Stimulated Phosphoprotein" (VASP) [12], which is, in turn, involved in "Breakpoint Cluster Region-Abelson" (BCR-ABL) signaling, a pathway reported to contribute to the production of tumors among patients with chronic myeloid leukemia [12]. PCDHGC3 is indirectly related to leukemia through the "Growth Factor Independent 1 Transcriptional Repressor" [13], SHP2 [14] and "Neural Cell Adhesion Molecule 1" [15].

Breast Cancer Estrogen Receptor dataset: The AUCTSP method has selected "Estrogen Receptor Alpha" (ESR1) and "Cathepsin C" (CTSC) as the most informative pair of genes for diagnosing estrogen receptor breast cancer. ESR1 amplification has been reported in benign as well as precancerous breast diseases [16]. Thus, ESR1 seems to be a possible biomarker for very early stage genetic changes for various kinds of breast cancers [16]. CTSC on the other hand, is up-regulated in "pII" cells in the collection of Michigan Cancer Foundation-7 [17], and is highly correlated with ESR1 [18]. The TSP method selected 5 pairs of genes with the same highest TSP score: ESR1 and "ETS2 Repressor Factor" (ERF), ESR1 and "Mucin 2" (MUC2), ESR1 and "Janus Kinase 3" (JAK3), ESR1 and "G Protein Subunit Beta 3" (GNB3), and ESR1 and "Histodyl-TRNA Synthetase 2" (HARS2). ERF is indirectly related to breast cancer, as it is down-regulated by estradiol (E2) which might increase the transcriptional activity of genes in breast cancer-related pathways [19]. MUC2 expression is known to be significantly involved with the ER status [20]. The JAK3 protein coding gene is mostly associated with immune system diseases and is known to be a major regulator for PLD2 and cell invasion [21]. GNB3 does not seem to be related to breast cancer per se [22], but it seems to play a role in bone metastasis in breast cancer patients [23]. HARS2 has not been reported to be related to breast cancer.

Breast Cancer Lymph Node dataset: The AUCTSP method selected the "Bacteriophage P1 Cre Recombinase Protein" (BP1CR) and "Glycophorin B (MNS Blood Group)" (GYPB) as the most informative pair of genes for diagnosing lymph node breast cancer. The TSP method selected BP1CR and "Keratin 31" (KRT31) from the same dataset as the top scoring pair. BP1CR is a genetically modified site-specific mutated gene used in genetic engineering for more precise control of the specific gene function and/or targeting of particular cell types [24, 25]. Therefore, we have considered the second most informative pair of genes given by AUCTSP and TSP. The AUCTSP method selected "Activin A Receptor Type 1B" (ACVR1B) and "Glycophorin B" (MNS Blood Group)" (GYPB) as the second most informative pair of genes for diagnosing lymph node breast cancer. ACVR1B has been well known to be an inhibition or stimulation factor in cell growth [26]. In the case of breast cancer, ACVR1B has a growth inhibitory effect [26]. ACVR1B has been associated with ER status, whereas ER positive cells were responsive to Activin A, while ER negative cells where resistant to it [26]. Implicating genes for the selected GYPB are: P53, PIK3CA, KRAS, AKT1 and EPCAM [27]. P53 mutations are known to be very common genetic changes responsible for forming tumorous tissues in the human body [28]. In breast cancer this situation gets even worse as the P53 mutations

get associated with more aggressive diseases [28]. The possibility that P53 mutations may affect the biological behavior in breast cancer has been known for a long time [29]. The PI3KCA/AKT pathway has also been known as an important biomarker for measuring breast cancer aggressiveness [30]. The TSP method selected ACVR1B and "Fatty Acid Binding Protein 3" (FABP3) from the same dataset as diagnostic biomarkers. There is some indication that the FABP protein family is involved in breast cancer [31, 32].

Diffuse Large B-Cell Lymphoma dataset: The AUCTSP method selected "Prostaglandin E Receptor 4" (PTGER4) and "RNA Polymerase II Subunit J" (POLR2J) as the most informative genes for diagnosing Diffuse Large B-cell lymphoma. PTGER4 has been known as a negative feedback regulator of the B-cell receptor (BCR) [33]. Most B-cell lymphomas are dependent on BCR signals for their survival [33]. Thus, discovering genes that control the BCR-mediated propagation is an important task in improving the quality of B-cell lymphoma therapy [33]. PTGER4 has been shown to be down-regulated in human B-cell lymphoma [33]. POLR2J is affected by the mixed-lineage leukemia gene (MLL) [34]. Chromosomal translocations of the MLL gene have direct effect on diffuse large B-cell lymphoma by mediating "Methylate Histone H3 on Lysine 4" [35]. The TSP method selected "Phosophodiesterase 4B" (PDE4B) and "G Protein-Coupled Receptor 12" (GPR12) from the same dataset as diagnostic biomarkers. Increased expression levels of PDE4B are a possible indication of B-lymphoid malignancies [36] while GPR12 has been shown to affect B-cell lymphoma/leukemia-2 (Bcl-2) regulation, and may have a role in cell survival [37].

DLBCL Follicular Lymphoma dataset: AUCTSP selected the "Fc Fragment of IgG Receptor Ia" (FCGR1A) and "Neogenin 1" (NEO1) as the most informative pair of genes for diagnosing DLBCL Follicular lymphoma. Three of the known classes of "Fc gamma Receptors" are expressed in human cells and the polymorphonuclear cells only happen to express the low-affinity receptor classes Fcγ Receptor II and Fcγ Receptor III [38]. The high-affinity receptor class, Fcγ Receptor I, only expresses under certain circumstances such as various malignancies or hematological disorders [38]. NEO1 has been reported to be affecting DLBCL follicular lymphoma through "Tumor Necrosis Factor" [39], "Interleukin 2" [40], "P53" [41] and "NOTCH 1" [42]. The TSP method selected "Small Nuclear Ribonucleoprotein Polypeptides B and B1" (SNRPB) and "Tyrosine 3-Monooxygenase/Tryptophan 5-Monooxygenase Activation Protein Zeta" (YWHAZ) from the same dataset as diagnostic biomarkers. SNRPB has been reported to have effect on DLBCL follicular lymphoma through "P53" [43], "MYC Proto-Oncogene", "BHLH Transcription Factor" [43] and "Cyclin Dependent Kinase Inhibitor 2A" [44]. "Zeta mediates" have been shown to be highly regulated in DLBCL lymph nodes compared to normal lymph nodes [45].

Colon Cancer dataset: The AUCTSP method selected "Heterogeneous Nuclear Ribonucleoprotein A1" (HNRNPA1) and "Myosin Heavy Polypeptide 9" (MYH9) as the most informative pair of genes for diagnosing colon cancer. HNRNPA1 expression is highly correlated with "Colon Cancer Associated Transcript 1" (CCAT1) [46] which is deregulated in several cancers such as colon cancer [46]. MYH9 is a downstream effector of the proprotein convertases involved in "Human Colon Carcinoma HT-29" cells [47]. The TSP method selected "Vasoactive Intestinal Peptide" (VIP) and "Aspartyl-TRNA Synthetase" (DARS) as the top scoring pair. Although the VIP expression levels have been reported to be significantly higher or lower among subjects with colon cancer compared to healthy subjects [48], DARS has not been reported to be associated with colon cancer.

Prostate Cancer dataset: The AUCTSP method selected "NUMB Endocytic Adaptor Protein" (NUMB) and "Complement Factor D" (CFD) as the top scoring pair of genes for diagnosing prostate cancer. NUMB has been reported to control prostate cancer tumors and has been used as a biomarker for the detection of prostate cancer [49]. CFD has been reported as the upstream effector of the kallikrein family [50], which are, in turn, highly expressed in prostate cancer [50]. The TSP method selected "Enolase 1" (ENO1) and CFD as diagnostic biomarkers for prostate cancer. Sequence analysis of ENO1 has revealed a high homology with "c-myc Promoter-binding Protein 1" (MBP-1) [51] and it is MBP-1 that has been reported to have an important role in blocking the growth of cancerous cells in prostate cancer [52].

References

1. Maines-Bandiera, S., Woo, M.M., Borugian, M., Molday, L.L., Hii, T., Gilks, B., Leung, P.C., Molday, R.S., Auersperg, N.: Oviductal glycoprotein (ovgp1, muc9): a differentiation-based mucin present in serum of women with ovarian cancer. International Journal of Gynecological Cancer **20**(1), 16–22 (2010)
2. Ravikumar, S., Perez-Liz, G., Del Vale, L., Soprano, D.R., Soprano, K.J.: Insulin receptor substrate-1 is an important mediator of ovarian cancer cell growth suppression by all-trans retinoic acid. Cancer research **67**(19), 9266–9275 (2007)
3. Li, S.-L., Ye, F., Cai, W.-J., Hu, H.-D., Hu, P., Ren, H., Zhu, F.-F., Zhang, D.-Z.: Quantitative proteome analysis of multidrug resistance in human ovarian cancer cell line. Journal of cellular biochemistry **109**(4), 625–633 (2010)
4. Talekar, M., Ouyang, Q., Goldberg, M.S., Amiji, M.M.: Cosilencing of pkm-2 and mdr-1 sensitizes multidrug-resistant ovarian cancer cells to paclitaxel in a murine model of ovarian cancer. Molecular cancer therapeutics **14**(7), 1521–1531 (2015)

5. Tinelli, A., Vergara, D., Martignago, R., Leo, G., Malvasi, A., Tinelli, R., Marsigliante, S., Maffia, M., Lorusso, V.: Ovarian cancer biomarkers: a focus on genomic and proteomic findings. Current genomics **8**(5), 335–342 (2007)
6. Mazurek, S.: Pyruvate kinase type m2: a key regulator of the metabolic budget system in tumor cells. The international journal of biochemistry & cell biology **43**(7), 969–980 (2011)
7. Golub, T.R., Slonim, D.K., Tamayo, P., Huard, C., Gaasenbeek, M., Mesirov, J.P., Coller, H., Loh, M.L., Downing, J.R., Caligiuri, M.A., *et al.*: Molecular classification of cancer: class discovery and class prediction by gene expression monitoring. science **286**(5439), 531–537 (1999)
8. Wolgast, L.R., Cannizzarro, L.A., Ramesh, K., Xue, X., Wang, D., Bhattacharyya, P.K., Gong, J.Z., McMahon, C., Albanese, J.M., Sunkara, J.L., *et al.*: Spectrin isoforms: differential expression in normal hematopoiesis and alterations in neoplastic bone marrow disorders. American journal of clinical pathology **136**(2), 300–308 (2011)
9. Gorman, E.B., Chen, L., Albanese, J., Ratech, H.: Patterns of spectrin expression in b-cell lymphomas: loss of spectrin isoforms is associated with nodule-forming and germinal center-related lymphomas. Modern Pathology **20**(12), 1245 (2007)
10. Sastry, S.K., Elferink, L.A.: Checks and balances: interplay of rtks and ptps in cancer progression. Biochemical pharmacology **82**(5), 435–440 (2011)
11. Bentires-Alj, M., Paez, J.G., David, F.S., Keilhack, H., Halmos, B., Naoki, K., Maris, J.M., Richardson, A., Bardelli, A., Sugarbaker, D.J., *et al.*: Activating mutations of the noonan syndrome-associated shp2/ptpn11 gene in human solid tumors and adult acute myelogenous leukemia. Cancer research **64**(24), 8816–8820 (2004)
12. Bernusso, V.A., Machado-Neto, J.A., Pericole, F.V., Vieira, K.P., Duarte, A.S., Traina, F., Hansen, M.D., Saad, S.T.O., Barcellos, K.S.: Imatinib restores vasp activity and its interaction with zyxin in bcr–abl leukemic cells. Biochimica et Biophysica Acta (BBA)-Molecular Cell Research **1853**(2), 388–395 (2015)
13. Zhan, R., Wu, S.-Q., Huang, H.-B., Huang, S.-L., Lin, J.: Gfi-1 expression in leukemia patients and inhibitory effects of lentiviral vector mediated silence of gfi-1 gene on proliferation in k562 cells. Zhongguo shi yan xue ye xue za zhi **18**(4), 849–854 (2010)
14. Xu, R., Yu, Y., Zheng, S., Zhao, X., Dong, Q., He, Z., Liang, Y., Lu, Q., Fang, Y., Gan, X., *et al.*: Overexpression of shp2 tyrosine phosphatase is implicated in leukemogenesis in adult human leukemia. Blood **106**(9), 3142–3149 (2005)
15. Ju, X., Peng, M., Xu, X., Lu, S., Li, Y., Ying, K., Xie, Y., Mao, Y., Xia, F.: Expression of cell adhesion molecules in acute leukemia cell. Zhonghua xue ye xue za zhi= Zhonghua xueyexue zazhi **23**(11), 581–584 (2002)
16. Holst, F., Stahl, P.R., Ruiz, C., Hellwinkel, O., Jehan, Z., Wendland, M., Lebeau, A., Terracciano, L., Al-Kuraya, K., Jänicke, F., *et al.*: Estrogen receptor alpha (esr1) gene amplification is frequent in breast cancer. Nature genetics **39**(5), 655 (2007)
17. Luqmani, Y., Al Azmi, A., Al Bader, M., Abraham, G., El Zawahri, M.: Modification of gene expression induced by sirna targeting of estrogen receptor α in mcf7 human breast cancer cells. International journal of oncology **34**(1), 231–242 (2009)
18. Chan, H.-S., Chang, S.-J., Wang, T.-Y., Ko, H.-J., Lin, Y.-C., Lin, K.-T., Chang, K.-M., Chuang, Y.-J.: Serine protease prss23 is upregulated by estrogen receptor α and associated with proliferation of breast cancer cells. PLoS One **7**(1), 30397 (2012)
19. Frasor, J., Danes, J.M., Komm, B., Chang, K.C., Lyttle, C.R., Katzenellenbogen, B.S.: Profiling of estrogen up-and down-regulated gene expression in human breast cancer cells: insights into gene networks and pathways underlying estrogenic control of proliferation and cell phenotype. Endocrinology **144**(10), 4562–4574 (2003)
20. Rakha, E.A., Boyce, R.W., El-Rehim, D.A., Kurien, T., Green, A.R., Paish, E.C., Robertson, J.F., Ellis, I.O.: Expression of mucins (muc1, muc2, muc3, muc4, muc5ac and muc6) and their prognostic significance in human breast cancer. Modern Pathology **18**(10), 1295 (2005)
21. Ye, Q., Kantonen, S., Gomez-Cambronero, J.: Serum deprivation confers the mda-mb-231 breast cancer line with an egfr/jak3/pld2 system that maximizes cancer cell invasion. Journal of molecular biology **425**(4), 755–766 (2013)
22. Krippl, P., Langsenlehner, U., Renner, W., Yazdani-Biuki, B., Wolf, G., Wascher, T.C., Paulweber, B., Samonigg, H.: The 825c¿ t polymorphism of the g-protein beta-3 subunit gene (gnb3) and breast cancer. Cancer letters **206**(1), 59–62 (2004)
23. Clar, H., Langsenlehner, U., Krippl, P., Renner, W., Leithner, A., Gruber, G., Hofmann, G., Yazdani-Biuki, B., Langsenlehner, T., Windhager, R.: A polymorphism in the g protein β3-subunit gene is associated with bone metastasis risk in breast cancer patients. Breast cancer research and treatment **111**(3), 449–452 (2008)
24. Vargo-Gogola, T., Rosen, J.M.: Modelling breast cancer: one size does not fit all. Nature reviews. Cancer **7**(9), 659 (2007)
25. Wierzbicki, A., Kendall, M., Abremski, K., Hoess, R.: A mutational analysis of the bacteriophage p1 recombinase cre. Journal of molecular biology **195**(4), 785–794 (1987)
26. Risbridger, G.P., Schmitt, J.F., Robertson, D.M.: Activins and inhibins in endocrine and other tumors. Endocrine Reviews **22**(6), 836–858 (2001)
27. Stelzer, G., Plaschkes, I., Oz-Levi, D., Alkelai, A., Olender, T., Zimmerman, S., Twik, M., Belinky, F., Fishilevich, S., Nudel, R., *et al.*: Varelect: the phenotype-based variation prioritizer of the genecards suite. BMC genomics **17**(2), 444 (2016)
28. Gasco, M., Shami, S., Crook, T.: The p53 pathway in breast cancer. Breast Cancer Research **4**(2), 70 (2002)
29. Mazars, R., Spinardi, L., BenCheikh, M., Simony-Lafontaine, J., Jeanteur, P., Theillet, C.: p53 mutations occur in aggressive breast cancer. Cancer research **52**(14), 3918–3923 (1992)
30. Tserga, A., Chatziandreou, I., Michalopoulos, N.V., Patsouris, E., Saetta, A.A.: Mutation of genes of the pi3k/akt pathway in breast cancer supports their potential importance as biomarker for breast cancer aggressiveness. Virchows Archiv **469**(1), 35–43 (2016)
31. Li, H., Lu, Q., Dong, L., Xue, H., Zhou, H., Yang, H.: Expression of fatty acid binding protein in human breast cancer tissues. Xi bao yu fen zi mian yi xue za zhi= Chinese journal of cellular and molecular immunology **23**(4), 312–316 (2007)
32. Buhlmann, C., Börchers, T., Pollak, M., Spener, F.: Fatty acid metabolism in human breast cancer cells (mcf7) transfected with heart-type fatty acid binding protein. Molecular and cellular biochemistry **199**(1-2), 41–48 (1999)
33. Murn, J., Alibert, O., Wu, N., Tendil, S., Gidrol, X.: Prostaglandin e2 regulates b cell proliferation through a candidate tumor suppressor, ptger4. Journal of Experimental Medicine **205**(13), 3091–3103 (2008)
34. Wang, P., Lin, C., Smith, E.R., Guo, H., Sanderson, B.W., Wu, M., Gogol, M., Alexander, T., Seidel, C., Wiedemann, L.M., *et al.*: Global analysis of h3k4 methylation defines mll family member targets and points to a role for mll1-mediated h3k4 methylation in the regulation of transcriptional initiation by rna polymerase ii. Molecular and cellular biology **29**(22), 6074–6085 (2009)
35. Morin, R.D., Mendez-Lago, M., Mungall, A.J., Goya, R., Mungall, K.L., Corbett, R., Johnson, N.A., Severson, T.M., Chiu, R., Field, M., *et al.*: Frequent mutation of histone modifying genes in non-hodgkin lymphoma. Nature **476**(7360), 298 (2011)
36. Smith, P.G., Wang, F., Wilkinson, K.N., Savage, K.J., Klein, U., Neuberg, D.S., Bollag, G., Shipp, M.A., Aguiar, R.C.: The phosphodiesterase pde4b limits camp-associated pi3k/akt–dependent apoptosis in diffuse large b-cell lymphoma. Blood **105**(1), 308–316 (2005)
37. Lu, X., Zhang, N., Meng, B., Dong, S., Hu, Y.: Involvement of gpr12 in the regulation of cell proliferation and survival. Molecular and cellular biochemistry **366**(1-2), 101–110 (2012)
38. Niitsu, N., Hayama, M., Okamoto, M., Khori, M., Higashihara, M., Tamaru, J.-i., Hirano, M.: Phase i study of rituximab-chop regimen in combination with granulocyte colony-stimulating factor in patients with follicular lymphoma. Clinical cancer research **10**(12), 4077–4082 (2004)
39. Wang, S.S., Cozen, W., Cerhan, J.R., Colt, J.S., Morton, L.M., Engels, E.A., Davis, S., Severson, R.K., Rothman, N., Chanock, S.J., *et al.*: Immune mechanisms in non–hodgkin lymphoma: Joint effects of the tnf g308a and il10 t3575a polymorphisms with non–hodgkin lymphoma risk factors. Cancer Research **67**(10), 5042–5054 (2007)
40. Fabre-Guillevin, E., Tabrizi, R., Coulon, V., Monnereau, A., Eghbali,

H., Soubeyran, I., Soubeyran, P.: Aggressive non-hodgkin's lymphoma: concomitant evaluation of interleukin-2, soluble interleukin-2 receptor, interleukin-4, interleukin-6, interleukin-10 and correlation with outcome. Leukemia & lymphoma **47**(4), 603–611 (2006)

41. Weinkauf, M., Christopeit, M., Hiddemann, W., Dreyling, M.: Proteome-and microarray-based expression analysis of lymphoma cell lines identifies a p53-centered cluster of differentially expressed proteins in mantle cell and follicular lymphoma. Electrophoresis **28**(23), 4416–4426 (2007)
42. Bohn, O., Maeda, T., Filatov, A., Lunardi, A., Pandolfi, P.P., Teruya-Feldstein, J.: Utility of lrf/pokemon and notch1 protein expression in the distinction between nodular lymphocyte-predominant hodgkin lymphoma and classical hodgkin lymphoma. International journal of surgical pathology **22**(1), 6–11 (2014)
43. Weinkauf, M., Christopeit, M., Hiddemann, W., Dreyling, M.: Proteome-and microarray-based expression analysis of lymphoma cell lines identifies a p53-centered cluster of differentially expressed proteins in mantle cell and follicular lymphoma. Electrophoresis **28**(23), 4416–4426 (2007)
44. Sánchez-Aguilera, A., Sánchez-Beato, M., Garcıa, J.F., Prieto, I., Pollan, M., Piris, M.A.: p14 arf nuclear overexpression in aggressive b-cell lymphomas is a sensor of malfunction of the common tumor suppressor pathways. Blood **99**(4), 1411–1418 (2002)
45. Maxwell, S.A., Li, Z., Jaya, D., Ballard, S., Ferrell, J., Fu, H.: 14-3-3ζ mediates resistance of diffuse large b cell lymphoma to an anthracycline-based chemotherapeutic regimen. Journal of Biological Chemistry **284**(33), 22379–22389 (2009)
46. Zhou, B., Wang, Y., Jiang, J., Jiang, H., Song, J., Han, T., Shi, J., Qiao, H.: The long noncoding rna colon cancer-associated transcript-1/mir-490 axis regulates gastric cancer cell migration by targeting hnrnpa1. IUBMB life **68**(3), 201–210 (2016)
47. Scamuffa, N., Metrakos, P., Calvo, F., Khatib, A.-M.: Identification of the myosin heavy polypeptide 9 as a downstream effector of the proprotein convertases in the human colon carcinoma ht-29 cells. Proprotein Convertases, 207–215 (2011)
48. Shah, H., McPherson, K., Mansour, A., Ramirez-Santrich, C., Nassiri, M., Shahda, S.: Neuroendocrine differentiation of a primary braf mutant colon cancer in a patient with a history of hairy cell leukemia. Clinical colorectal cancer **15**(4), 235–239 (2016)
49. Flores, A.N., McDermott, N., Meunier, A., Marignol, L.: Numb inhibition of notch signalling as a therapeutic target in prostate cancer. Nature Reviews Urology **11**(9), 499–507 (2014)
50. Yousef, G.M., Kopolovic, A.D., Elliott, M.B., Diamandis, E.P.: Genomic overview of serine proteases. Biochemical and biophysical research communications **305**(1), 28–36 (2003)
51. Giallongo, A., Feo, S., Moore, R., Croce, C.M., Showe, L.C.: Molecular cloning and nucleotide sequence of a full-length cdna for human alpha enolase. Proceedings of the National Academy of Sciences **83**(18), 6741–6745 (1986)
52. Ghosh, A.K., Steele, R., Ray, R.B.: c-myc promoter-binding protein 1 (mbp-1) regulates prostate cancer cell growth by inhibiting mapk pathway. Journal of Biological Chemistry **280**(14), 14325–14330 (2005)
