# Supplementary material for: AUCTSP: an improved biomarker gene pair class predictor
Source: BMC Bioinformatics. 2018 Jun 26;19:244. doi: 10.1186/s12859-018-2231-1 (PMC6020231; doi:10.1186/s12859-018-2231-1)
Supplement: Supplementary file 2 — Histograms of the selected genes. The histograms of all the genes selected by AUCTSP and TSP are given. (PDF 294 kb) [file 12859_2018_2231_MOESM2_ESM.pdf]

Additional file 2 - Histograms of the selected genes

- Figure 1 represents the histograms of the genes selected by AUCTSP and TSP from the Ovarian Cancer dataset.
- Figure 2 represents the histograms of the genes selected by AUCTSP and TSP from the Leukemia Cancer dataset.
- Figure 3 represents the histograms of the genes selected by AUCTSP and TSP from the Breast-ER Cancer dataset.
- Figure 4 represents the histograms of the genes selected by AUCTSP and TSP from the Breast-LN Cancer dataset.
- Figure 5 represents the histograms of the genes selected by AUCTSP and TSP from the DLBCL dataset.
- Figure 6 represents the histograms of the genes selected by AUCTSP and TSP from the DLBCL-FL dataset.
- Figure 7 represents the histograms of the genes selected by AUCTSP and TSP from the Colon Cancer dataset.
- Figure 8 represents the histograms of the genes selected by AUCTSP and TSP from the Prostate Cancer dataset.

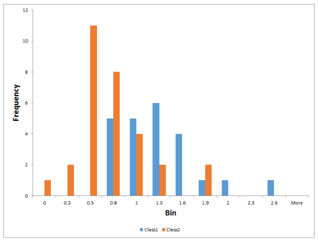

(a) IRS1

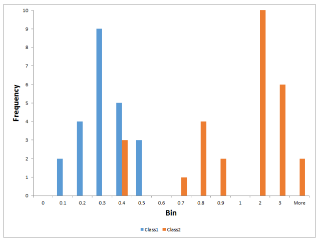

(b) OVGP1

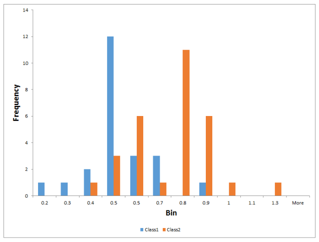

(c) PKM2

Figure 1: Histograms of the genes selected from the Ovarian Cancer dataset

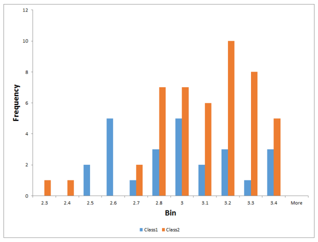

(a) ARHGAP45

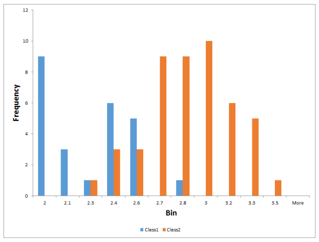

(b) SPTAN1

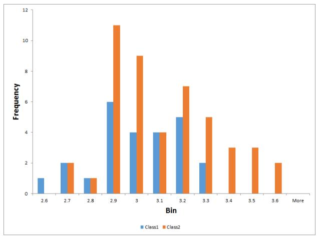

(c) PCDHGC3

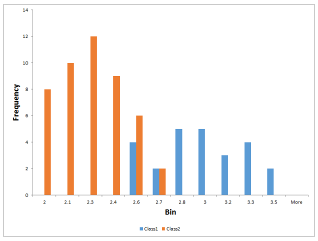

(d) CD33

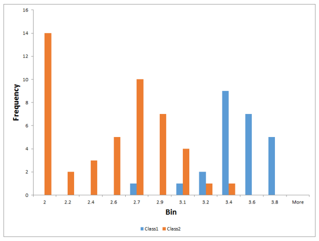

(e) ZYX

Figure 2: Histograms of the genes selected from the Leukemia Cancer dataset

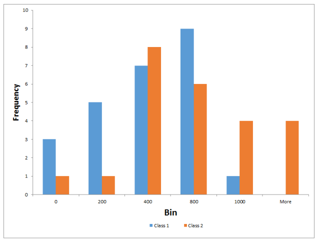

(a) MUC2

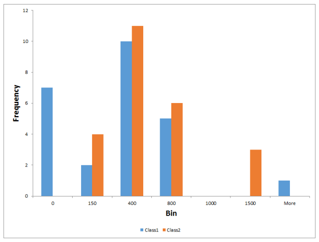

(b) JAK3

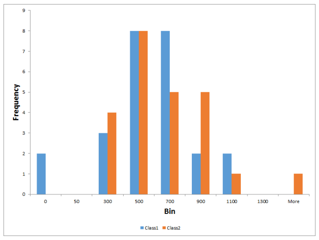

(c) ERF

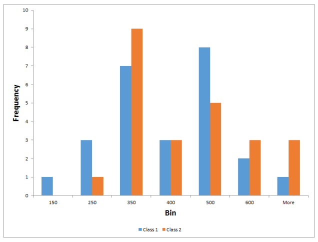

(d) HARS2

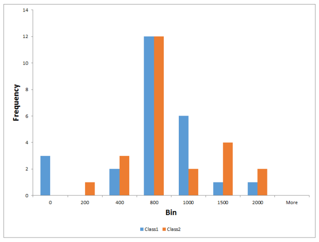

(e) GNB3

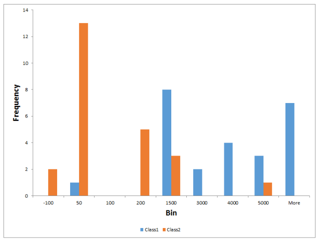

(f) ESR1

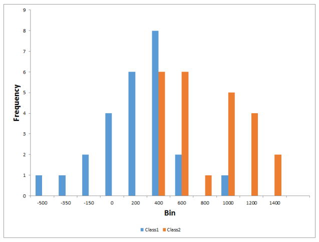

(g) CTSC

Figure 3: Histograms of the genes selected from the Breast-ER Cancer dataset

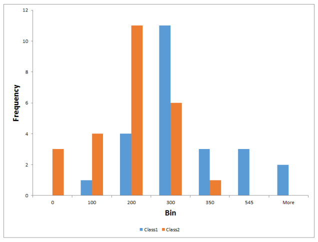

(a) GYPB

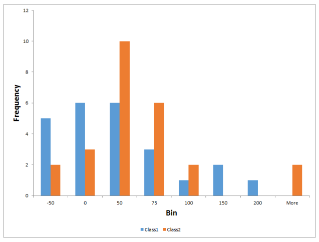

(b) FABP3

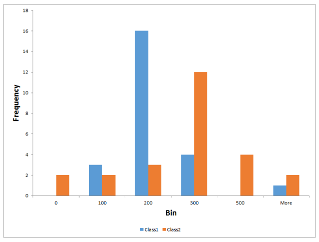

(c) ACVR1B

Figure 4: Histograms of the genes selected from the Breast-LN Cancer dataset

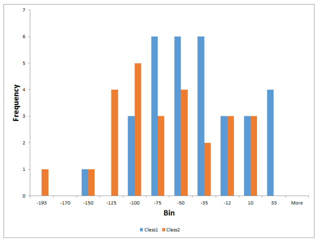

(a) POLR2J

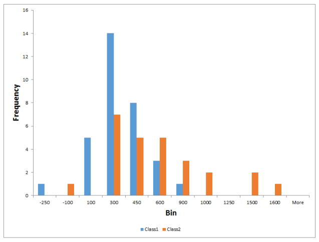

(b) PDE4B

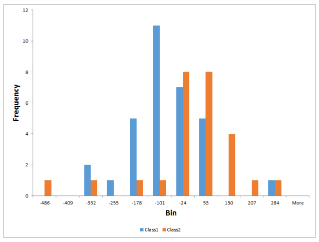

(c) PTGER4

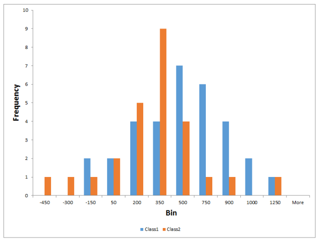

(d) GRP12

Figure 5: Histograms of the genes selected from the DLBCL dataset

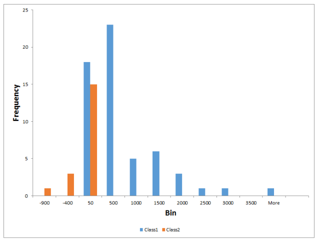

(a) YWHAZ

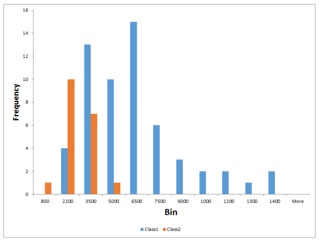

(b) FCGR1A

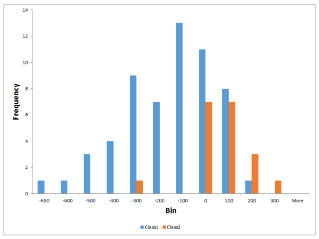

(c) NEO1

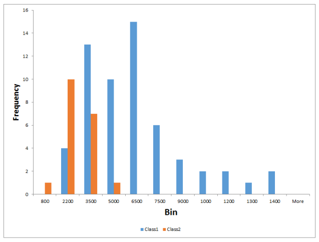

(d) SNRPB

Figure 6: Histograms of the genes selected from the DLBCL-FL dataset

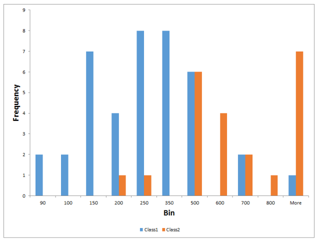

(a) MYH9

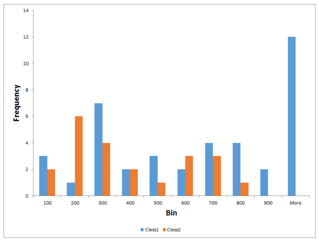

(b) HNRNPA1

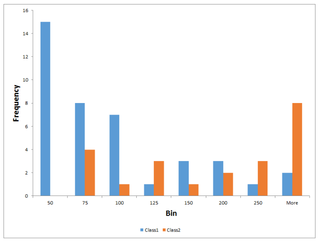

(c) VIP

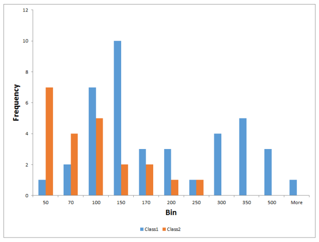

(d) DARS

Figure 7: Histograms of the genes selected from the Colon Cancer dataset

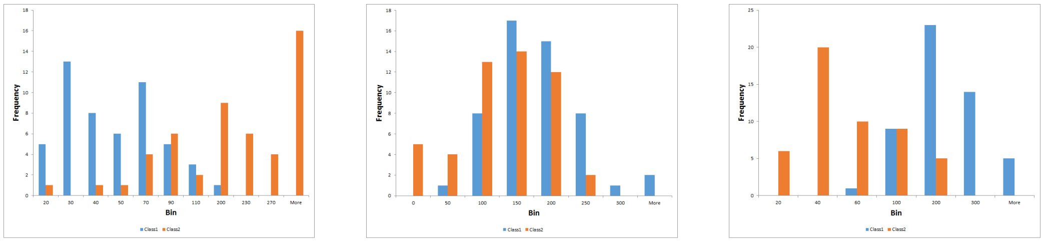

(a) CFD (b) ENO1 (c) NUMB

Figure 8: Histograms of the genes selected from the Prostate Cancer dataset
